# Hyaluronic Acid Methacrylate Hydrogel-Modified Electrochemical Device for Adsorptive Removal of Lead(II)

**DOI:** 10.3390/bios12090714

**Published:** 2022-09-02

**Authors:** Nan Wang, Meghali Bora, Song Hao, Kai Tao, Jin Wu, Liangxing Hu, Jianjun Liao, Shiwei Lin, Michael S. Triantafyllou, Xiaogan Li

**Affiliations:** 1 School of Microelectronics, Dalian University of Technology, Dalian 116024, China; 2 Center for Environmental Sensing and Modeling (CENSAM) IRG, Singapore-MIT Alliance for Research and Technology (SMART) Centre, Singapore 138602, Singapore; 3 School of Optoelectronic Engineering and Instrumentation Science, Dalian University of Technology, Dalian 116024, China; 4 Ministry of Education Key Laboratory of Micro and Nano Systems for Aerospace, School of Mechanical Engineering, Northwestern Polytechnical University, Xi’an 710072, China; 5 State Key Laboratory of Optoelectronic Materials and Technologies and the Guangdong Province Key Laboratory of Display Material and Technology, School of Electronics and Information Technology, Sun Yat-sen University, Guangzhou 510275, China; 6 School of Electrical and Electronic Engineering, Nanyang Technological University, Singapore 639798, Singapore; 7 Key Laboratory of Agro-Forestry Environmental Processes and Ecological Regulation of Hainan Province, School of Ecological and Environmental Sciences, Hainan University, Haikou 570228, China; 8 School of Materials Science and Engineering, Hainan University, Haikou 570228, China; 9 Department of Mechanical Engineering, Massachusetts Institute of Technology, Cambridge, MA 02139, USA

**Keywords:** electrochemical device, hyaluronic acid methacrylate hydrogel, metal ion–amide complexation, electrochemical accumulation, lead removal

## Abstract

This paper presents the development of a compact, three-electrode electrochemical device functionalized by a biocompatible layer of hyaluronic acid methacrylate (HAMA) hydrogel for the adsorptive removal of detrimental lead (Pb(II)) ions in aqueous solutions. An adsorption mechanism pertaining to the observed analytical performance of the device is proposed and further experimentally corroborated. It is demonstrated that both the molecular interactions originating from the HAMA hydrogel and electrochemical accumulation originating from the electrode beneath contribute to the adsorption capability of the device. Infrared spectral analysis reveals that the molecular interaction is mainly induced by the amide functional group of the HAMA hydrogel, which is capable of forming the Pb(II)–amide complex. In addition, inductively coupled plasma mass spectrometric (ICP-MS) analysis indicates that the electrochemical accumulation is particularly valuable in facilitating the adsorption rate of the device by maintaining a high ion-concentration gradient between the solution and the hydrogel layer. ICP-MS measurements show that 94.08% of Pb(II) ions present in the test solution can be adsorbed by the device within 30 min. The HAMA hydrogel-modified electrochemical devices exhibit reproducible performance in the aspect of Pb(II) removal from tap water, with a relative standard deviation (RSD) of 1.28% (for *n* = 8). The experimental results suggest that the HAMA hydrogel-modified electrochemical device can potentially be used for the rapid, on-field remediation of Pb(II) contamination.

## 1. Introduction

Water contamination caused by toxic heavy metals has always been one of the greatest threats to public health due to the aversion of heavy metals to natural degradation. Such non-biodegradable properties enable heavy metals to move along the food chain, which eventually accumulate inside the human body, resulting in severe detrimental effects on human health. Among the different kinds of heavy metals, lead (Pb(II)) is considered the most hazardous contaminant owing to its strong toxicity. For instance, Pb(II) is capable of restraining the formation of hemoglobin, which is an essential component of red blood cells [1,2]. Other pathological symptoms, such as abdominal pain, arthralgia, anemia, and cognitive deficit, may also occur when exposure rises to a certain limit [3]. More worryingly, the intake of Pb(II) may cause permanent learning and behavioral disorders in infants and children [4,5,6]. Hence, the development of devices to detect Pb(II) has been of considerable interest to both academia and industry. Over the past decade, many researchers have reported a variety of successfully designed chemical/electrochemical [7,8,9,10], fluorescent [11,12,13], as well as biological devices [14,15,16] for Pb(II) detection. However, research on developing compact devices for the removal of Pb(II) is still scanty, although the importance of this research area has been reiterated in recent years because of the water crisis [17].

Conventional approaches to achieving the removal of Pb(II) from aqueous solutions include adsorption, chemical precipitation, ion exchange, reverse osmosis, coagulation, and membrane filtration. Among all these approaches, adsorption has proved to be the most practical method due to its simplicity of design and operation, high efficiency, and economical advantage [18,19,20]. Different kinds of polymer materials, such as poly(pyrrole methane) [21], poly(allylamine-co-methacrylamide-co-acrylic acid) [22], poly(*N*,*N*-dimethylacrylamide-co-2-hydroxyethyl methacrylate) [23], poly(acrylamide-co-itaconic acid) [24], melamine-formaldehyde-diaminohexane [25], poly(*trans*-aconitic acid/2-hydroxyethyl acrylate) [26], polyisoprene-*b*-polystyrene-*b*-poly(*N*,*N*-dimethylacrylamide) [27], nanochitosan/polyurethane/polypropylene glycol [28], etc., have been functionalized or directly utilized as effective adsorbents. Hyaluronic acid (HA), a carbohydrate polymer with repeated disaccharide units of glucuronic acid and N-acetylglucosamine alternatively linked by β-1,3 and β-1,4 glycosidic bonds, is widely present inside the human body (e.g., muscular connective tissues, epithelial tissues, and extracellular matrices). The excellent biocompatible, nontoxic, and biodegradable characteristics of HA permit it to be extensively adopted for clinical, surgical, and biomedical applications, such as (1) the formation of a surgical glue with a higher shear strength for tissue adhesion [29], (2) the development of an implantable macroporous scaffold with degradation regulatability to control tumor microenvironments [30], (3) the manufacture of a pH-triggered nanogel system for tumor-targeted drug delivery [31], and (4) the functionalization of a contact lens surface with improved wettability, water retention, and reduced protein binding [32], to name a few. However, the modification of HA for analytical devices for the adsorptive removal of heavy metal ions is yet to be explored.

In this work, we have developed an electrochemical device that incorporates a layer of methacrylated HA hydrogel for in situ adsorption of Pb(II) in solutions. The electrochemical device is configured to have three electrodes, i.e., one working electrode, one counter electrode, and one reference electrode. The primary polymer is chosen as HA and methacrylic anhydride (MA) is used as the precursor. The adsorption performance of the HAMA hydrogel-modified electrochemical device is theoretically analyzed and further comprehensively investigated through a series of experiments. The practical application of the HAMA hydrogel-modified electrochemical devices for Pb(II) removal from tap water is demonstrated. The use of electrochemical devices for the detection of heavy metal ions has been extensively studied in the past [7,8,9,10]. However, the utilization of electrochemical devices for the adsorptive removal of heavy metal ions has rarely been reported. This work paves the way for the development of compact electrochemical devices for in situ removal of heavy metal ions.

## 2. Materials and Methods

### 2.1. Chemicals and Reagents

The chemicals and reagents used throughout this study were of analytical grade. Standard Pb(II) and bismuth (Bi(II)) stock solutions (1000 mg/L) were purchased from Sigma-Aldrich and Merck, respectively. Ultrapure water (18.2 MΩ·cm) collected from a Milli-Q system was used to dilute the stock solutions. Acetate buffer (pH 4.6) was added into diluted Pb(II) solutions as a supporting electrolyte. HA (molecular weight, 1 MDa) was purchased from Samich (HK) Limited, China. Sodium chloride (NaCl), sodium hydroxide (NaOH), MA, and absolute ethanol were used without purification. Nafion 117 solution (5 wt% in a mixture of water and lower alcohols) was diluted by absolute ethanol. Photoinitiator Irgacure I2959 (I2959) was purchased from Ciba Specialty Chemicals, Switzerland.

### 2.2. Device Fabrication

**Figure 1:**
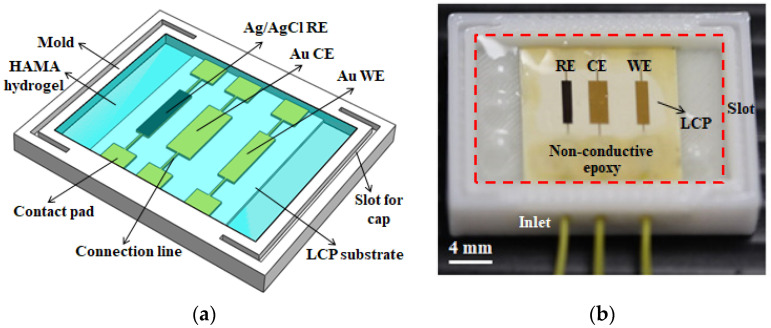
(**a**) Schematic drawing to illustrate the structure of the HAMA hydrogel-modified electrochemical device. (**b**) Photograph of the HAMA hydrogel-modified electrochemical device after packaging and modification, in which the cap on two sides of the polycarbonate mold is removed in order to make the swollen hydrogel visible. A transparent HAMA hydrogel layer is successfully formed on top of the electrochemical device inside the mold, which can be seen in the region marked by the dashed red rectangle.

As illustrated in Figure 1a, the electrochemical devices were fabricated via standard microfabrication techniques [33]. Initially, a liquid crystal polymer (LCP) sheet (ULTRALAM 3850, 100 µm) with copper cladding (18 µm) purchased from Rogers Corporation, USA, was cut into a 4-inch wafer size and used as a substrate for the devices. The cladding layer was removed by dipping the sheet in the copper etchant for 45 min, during which agitation was provided in order to facilitate the etching process. The sheet was then ultrasonically cleaned with acetone and deionized water to remove both organic and inorganic impurities. After drying with nitrogen gas, the sheet was thermally attached to a 4-inch silicon wafer using a photoresist (AZ 9260, 10 µm) as an adhesion layer. Thereafter, another layer of the photoresist (5 µm) was spin-coated on top of the LCP sheet and baked on a hotplate at 110 °C for 4 min. Upon the exposure of the photoresist under ultraviolet (UV, 365 nm, i-line) light followed by developing in the photoresist developer solution (AZ 400K), the patterns of the electrodes, connection lines, and contact pads were formed. After photolithography, a layer of chromium/gold (Cr/Au, 50/300 nm) was deposited using a magnetron sputtering system, in which the Cr layer was used to improve adhesion between the Au layer and the substrate. The remaining photoresist together with the deposited material was completely removed through the lift-off process by dipping the wafer in acetone solution for 12 h. This Au layer served as both the working and counter electrodes for the electrochemical devices. By repeating the photolithography-deposition procedure with a different photomask, the reference electrodes of the electrochemical devices were constructed by a combined layer of silver/silver chloride (Ag/AgCl, 150/250 nm).

### 2.3. Device Packaging

The packaging of the electrochemical devices was performed by connecting three wires with one set of contact pads of the working, counter, and reference electrodes, respectively, using EPO-TEK^®^ H20E conductive epoxy (Epoxy Technology, Billerica, MA, USA). After mixing the resin part A and the hardener part B of the H20E conductive epoxy in a weight ratio of 1:1, the mixture was applied to each contact pad. Each device was then baked inside an oven at 80 °C for 3 h to solidify the conductive epoxy. The resistance of all three electrodes between the other set of contact pads and the wires was measured after the baking process. If the resistance of any electrode was 10% higher than the average value, that device was excluded from subsequent modification and experiment. This is due to the concern that a higher resistance value usually suggests bad connectivity of the Au layer, which can be caused by several factors, such as stripping of the metal connection line, a crack on the electrode or the contact pad, and peeling of the Au layer at certain locations. Thereafter, the EPO-TEK^®^ H70E non-conductive epoxy mixture (resin part A and hardener part B in a weight ratio of 1:1) was drop-casted on the devices to cover all the contact pads (refer to Figure 1b) in order to provide electrical insulation. The non-conductive epoxy was cured by baking inside an oven at 80 °C for 1.5 h. Each packaged device was then placed into a 3D-printed polycarbonate mold for further modification. The detailed dimensions of the mold are presented in Figure S1.

### 2.4. Device Modification

**Figure 2:**
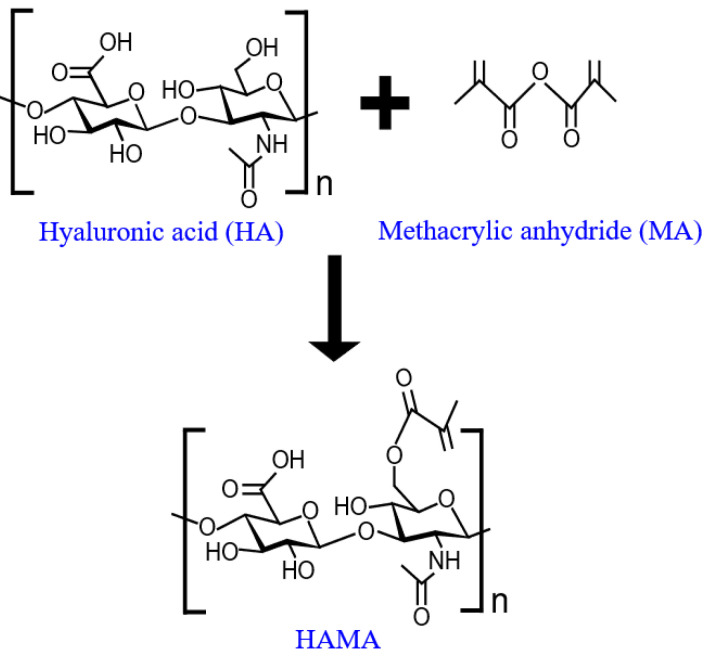
Schematic representation of the synthetic route of HAMA hydrogel.

Hyaluronic acid was modified with methacrylic anhydride and then photocrosslinked to obtain the HAMA hydrogel [34]. Briefly, the HA solution was prepared in ultrapure water and MA (20 mol/L) was added to it. The pH of the solution was adjusted to 8 using 1 M NaOH. After 2 h of reaction (as illustrated in Figure 2), the HAMA solution was incubated at 4 °C for 24 h and dialyzed (Spectra/Por 6 dialysis tubing, 10 kDa molecular weight cutoff) against 0.1 M NaCl solution, 25% (*v*/*v*) ethanol and ultrapure water for 48 h at room temperature. The modified HAMA solution was freeze-dried for 72 h and used to prepare the HAMA hydrogel. An amount of 1% (*w*/*v*) of HAMA was first dissolved in ultrapure water and then 1% (*w*/*w*) of I2959 photoinitiator (prepared in Nafion 117) was added to it and mixed thoroughly. Thereafter, each electrochemical device was contained in the polycarbonate mold, which had inlets for wires and cutout slots to hold a cap on both sides of the mold to keep the swollen hydrogel intact. The HAMA solution was spread uniformly over the device inside the mold without making any air bubbles and then exposed to UV light for 5 min to complete the crosslinking process. The HAMA hydrogel over the device was swollen in ultrapure water for 24 h to form a transparent gel layer, as shown in the dashed red rectangle in Figure 1b.

### 2.5. Investigation of Morphological and Chemical Structures of the Synthesized HAMA Hydrogel

The HAMA hydrogels were prepared using a similar approach to that mentioned earlier with slight modifications. The hydrogels were formed inside 3D-printed disc-shaped polycarbonate molds (internal diameter 18 mm, outer diameter 20 mm, and depth 2.5 mm). The HAMA solution together with the photoinitiator was spread uniformly into these molds and exposed to UV light for crosslinking. After crosslinking, the hydrogels were carefully removed from the molds and transferred to Petri dishes for swelling in ultrapure water. For the swelling capacity study, the weights of all the wet hydrogel samples were first recorded. Subsequently, the weights of all the dried hydrogels were measured upon baking the samples inside an oven at 50 °C for 6 h. For scanning electron microscopy (SEM) imaging, the hydrogel samples were quenched in liquid nitrogen for a few seconds and then immediately lyophilized for 72 h. The dried samples were coated with Au and imaged under an SEM microscope (JSM 6360A Jeol, Tokyo, Japan) at a 10 kV acceleration voltage. For Fourier transform infrared spectroscopy (FTIR) analysis, after 24 h of swelling, the ultrapure water was replaced by either a 0.1 M acetate buffer or 100 µg/L Pb(II) solution. Before conducting FTIR analysis, the hydrogel samples were dried inside an oven at 50 °C for 6 h. The FTIR experiments were carried out using a Nicolet iS10 FTIR Spectrometer (Thermo Fisher Scientific, Waltham, MA, USA) in a frequency range between 400 and 4000 cm^−1^. A total number of 32 scans with a resolution of 2 cm^−1^ were averaged for each spectrum.

### 2.6. Investigation of Adsorption Performance of the Modified Device

The adsorption performance of the HAMA hydrogel-modified electrochemical devices was evaluated by conducting a series of square-wave anodic stripping voltammetry (SWASV) in the diluted Pb(II) solutions. The output of the devices was recorded on a CHI 600 C electrochemical workstation (CH Instruments, Austin, TX, USA). The SWASV measurements were initiated by applying a deposition potential of −1.0 V to the working electrode for 120 s while the test solution was stirred (800 rpm). This deposition step was aimed at the collection of Pb(II) ions that were available in the vicinity of the working electrode. After an equilibration time of 2 s, the voltammograms were recorded under quiescent conditions in a potential range from −1.2 to 0 V with a frequency of 50 Hz, amplitude of 50 mV, and a step potential of 5 mV. This stripping step was proportionally related to the deposition step, i.e., the higher the stripping current, the more the amount of Pb(II) ions that were collected during the deposition step. Hence, a higher concentration of Pb(II) should be present in the test solution. Prior to the next measurement, a conditioning potential of −0.1 V was provided for 120 s in order to electrochemically clean the working electrode. Herein, all the potentials applied or measured during the SWASV experiments were with respect to the potential of the fabricated Ag/AgCl reference electrode.

### 2.7. Investigation of Adsorption Efficiency of the Modified Device

The adsorption rate of the HAMA hydrogel-modified electrochemical devices was determined by applying a deposition potential of −1.0 V (with respect to the Ag/AgCl reference electrode) for 5 min to the working electrode in a stirred (800 rpm) solution with 100 µg/L Pb(II). Thereafter, 5 mL of the test solution was transferred to a conical tube. The Pb(II) concentration in the solution was measured using an Agilent 7700 inductively coupled plasma mass spectrometry (ICP-MS) system (Agilent Technologies, Lexington, MA, USA). This series of experiments were repeated 8 times within 40 min, i.e., the Pb(II) concentration in each test solution was separately measured after a period of 5, 10, 15, 20, 25, 30, 35, and 40 min of adsorption. In order to further investigate the influence of electrochemical accumulation on the adsorption performance of the modified devices, another series of experiments were conducted with the same experimental procedure, except for applying the deposition potential, i.e., without the activation of electrochemical accumulation.

The applicability of the HAMA hydrogel-modified electrochemical devices was explored by testing the Pb(II) removal ability of the devices in tap water. Experiments were conducted by keeping the devices in tap water with 400 µg/L Pb(II) for 20 min with the activation of electrochemical accumulation. Thereafter, the Pb(II) concentration in the tap water was measured using the ICP-MS system. The removal efficiency of each device was calculated based on the concentration difference before and after the adsorption.

## 3. Results and Discussion

### 3.1. Characterization of the Synthesized HAMA Hydrogel

**Figure 3:**
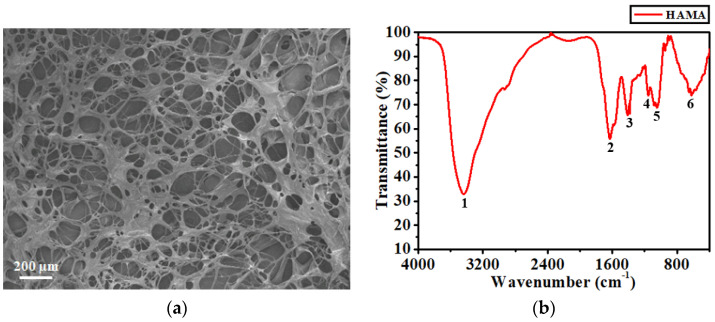
(**a**) Cross-sectional SEM image and (**b**) FTIR spectrum of the synthesized HAMA hydrogel. The numbers in (**b**) denote the prominent peaks detected by the FTIR analysis.

First, the swelling capacities of the synthesized HAMA hydrogels were evaluated using a gravimetrical approach. The weights of both the fully swollen and thermally dried hydrogel samples were separately determined. The results obtained are presented in Figure S2, where it can be observed that the average weight before swelling was ~8.17 mg. Once the dried samples had absorbed a sufficient amount of water to reach the equilibrium state of swelling (refer to Figure S3), the average weight dramatically increased to ~1526.23 mg, giving an average swelling ratio of 18,581%. This massive extent of swelling could be due to the superhydrophilic property of the HA. Thereafter, the morphology of the microscopic structure established inside the hydrogel layer was inspected using SEM. Figure 3a depicts the SEM image to illustrate the cross-sectional view of the synthesized HAMA hydrogel. It can be observed that a highly porous structure with an interconnected backbone was formed, in which most pores had either an oval or elongated bubble shape and their size varied from 20 to 200 µm in diameter. Such a high density of the porous structure also proved the considerable swelling capacity exhibited by the HAMA hydrogels.

The functional groups associated with the synthesized HAMA hydrogels were identified by FTIR analysis. The FTIR spectrum acquired is presented in Figure 3b, in which numbers (1 to 6) are used to denote the prominent peaks detected. Among all the peaks, peak 1 situated at 3436.34 cm^−1^ was much broader than the others, which could be due to the O-H or N-H stretching, considering that both stretching vibrations occurred at an overlapped frequency band between 3200 and 3600 cm^−1^ [35]. Peak 2 situated at 1628.07 cm^−1^ was likely contributed by the carbonyl (C=O) stretching rather than the N-H bending of the secondary amide group. This is because, for most secondary amides, the weak N-H bending adsorption often appears at the frequency band between 1500 to 1560 cm^−1^ [36]. Peak 3 situated at 1410.64 cm^−1^ corresponded to the vibration of the O-C=O group [37]. Peaks 4 and 5 situated at 1153.41 and 1046.36 cm^−1^ were related to the C-O and C-O-C stretching, respectively. Peak 6 situated at 650.68 cm^−1^ was a fingerprint peak of HA [37], which could have been caused by a combinational manner of bending vibrations.

### 3.2. Electrochemical Investigation of the HAMA Hydrogel-Modified Device

**Figure 4:**
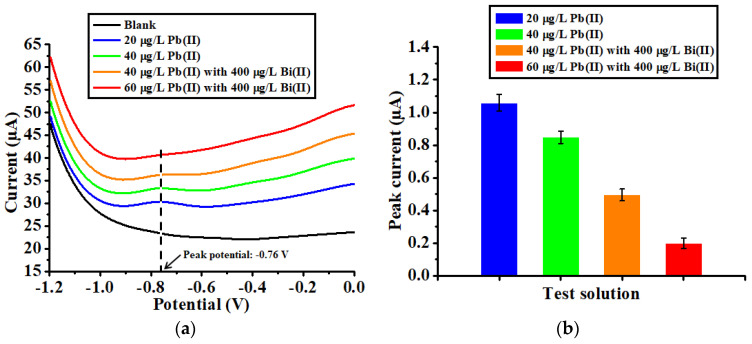
(**a**) Anodic stripping voltammograms and (**b**) the corresponding stripping peak currents recorded for the HAMA hydrogel-modified electrochemical devices in different test solutions.

To evaluate the adsorption performance of the HAMA hydrogel-modified electrochemical devices, a series of SWASV experiments were carried out. The magnitude of each stripping peak obtained in the SWASV experiments was measured with respect to its baseline. Initially, the response was recorded in a solution with 20 µg/L Pb(II) (blue line in Figure 4a), in which a clear stripping peak was observed close to a potential −0.76 V, with an average peak magnitude of 1.059 µA (refer to Figure 4b). When the Pb(II) concentration was increased to 40 µg/L, the device exhibited a smaller stripping peak (green line in Figure 4a) with a decreased average peak magnitude of 0.848 µA. To investigate whether such a decrease in the stripping peak was caused by the reduced alloying capability of the working electrode, 400 µg/L Bi(II) was added to the test solution. According to the literature [38,39,40], Bi is capable of forming low-melting-temperature alloys with heavy metals, which significantly facilitates the accumulation of metal ions by the working electrode in the course of the deposition. As shown by the orange line in Figure 4a, adding Bi(II) did not improve the accumulation of Pb(II) ions for the device, which was manifested by an even smaller stripping peak with a further decreased average peak magnitude of 0.496 µA. With 400 µg/L Bi(II) in the test solution, a subsequent increase in the Pb(II) concentration to 60 µg/L (red line in Figure 4a) resulted in an indistinguishable stripping peak with a tiny average peak magnitude of 0.199 µA. The experimental results imply that the developed HAMA hydrogel-modified electrochemical device is capable of adsorbing the Pb(II) ions that are available in the solution.

### 3.3. Adsorption Mechanism of the HAMA Hydrogel-Modified Device

**Figure 5:**
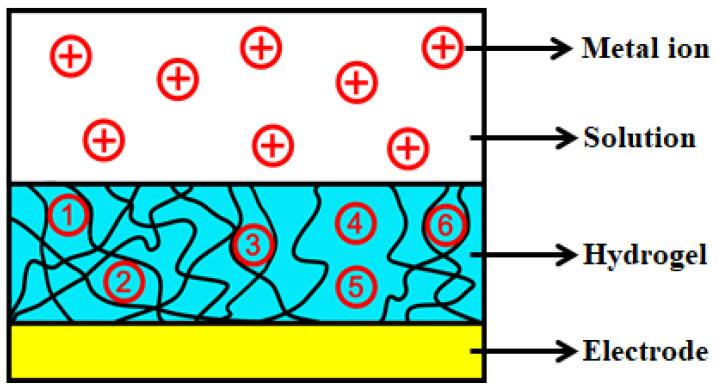
Schematic model to illustrate the adsorption mechanism of the HAMA hydrogel-modified electrochemical device. Red circles (⊕ and ①–⑥) represent heavy metal ions.

To explain the observed phenomena, a hypothesis pertaining to the adsorption mechanism of the HAMA hydrogel-modified electrochemical devices is proposed. A schematic model illustrating the mechanism is depicted in Figure 5, in which red circles (with a plus sign or number) represent the dissolved heavy metal ions in the solution. The network denoted by black solid lines above the electrode layer represents the microscopic porous structure of the HAMA hydrogel. First, free-moving metal ions in the solution migrated to the vicinity of the hydrogel under the effect of diffusion due to a high concentration gradient established between the solution and the hydrogel. Thereafter, the metal ions penetrated the hydrogel and interacted with the functional groups of the hydrogel’s polymer chain. Some of the metal ions (e.g., No. 1, 2, 3, and 6 in Figure 5) were directly captured by the polymer chain due to the molecular interaction.

On the other hand, some other metal ions (e.g., No. 4 and 5 in Figure 5) reached the surface of the electrode by moving through the holes formed in the porous hydrogel. These ions were further captured by the electrode due to the electrochemical accumulation (M^+^ + e^−^→M) triggered by the applied deposition potential. We suspect that most of the metal ions were collected by the hydrogel’s polymer chain considering the above electrochemical investigation. If the dominant adsorption was contributed by the electrochemical accumulation, a relatively larger stripping peak should have been observed from the voltammograms when the SWASV experiment was conducted in solutions with a higher concentration. However, without the electrochemically induced adsorption, the collection efficiency of the device should be adversely affected since the high concentration gradient between the solution and the hydrogel could not be continuously maintained. Therefore, we attribute the promising adsorption capability of the HAMA hydrogel-modified device toward the Pb(II) ions to a combined effect of the molecular interaction and electrochemical accumulation.

### 3.4. Validation of the Molecular Interaction

**Figure 6:**
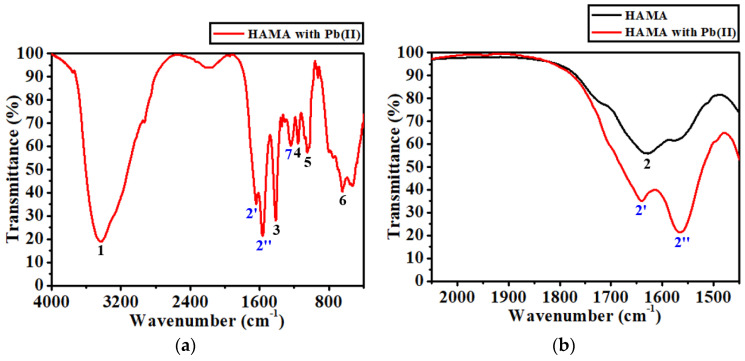
(**a**) FTIR spectrum of the HAMA hydrogel soaked in a solution of 100 µg/L Pb(II), in which the numbers 2′, 2″, 7 (in blue) denote the new peaks. (**b**) Comparison of FTIR spectra in a frequency band from 2050 to 1450 cm^−1^ between the pure HAMA hydrogel and the one soaked in Pb(II) solution.

The proposed hypothesis, specifically the molecular interaction, was validated by analyzing the FTIR spectrum obtained from a HAMA hydrogel sample soaked in a Pb(II) solution, as shown in Figure 6a. A comparison of all of the prominent peak positions in terms of wavenumber (cm^−1^) between the pure HAMA hydrogel and the Pb(II)-soaked HAMA hydrogel is listed in Table 1. It was found that peaks 1, 3, 4, 5, and 6 in both spectra were situated at similar frequency bands. A new peak 7 emerged at 1237.61 cm^−1^ for the Pb(II)-soaked HAMA hydrogel, which could have been due to either C-O or C-N stretching. In addition, the original peak 2 (corresponding to the carbonyl stretching of the amide group) for the pure HAMA hydrogel separated into two peaks, i.e., peak 2′ situated at 1640.03 cm^−1^ and peak 2″ situated at 1565.72 cm^−1^ for the Pb(II)-soaked HAMA hydrogel, as depicted in Figure 6b. The significant shift of peak 2 implies that the Pb(II) ions mainly interact with the amide group of the HAMA hydrogel. We attribute such a molecular interaction to the effect of the Pb(II)–amide complexation.

**Figure 7:**
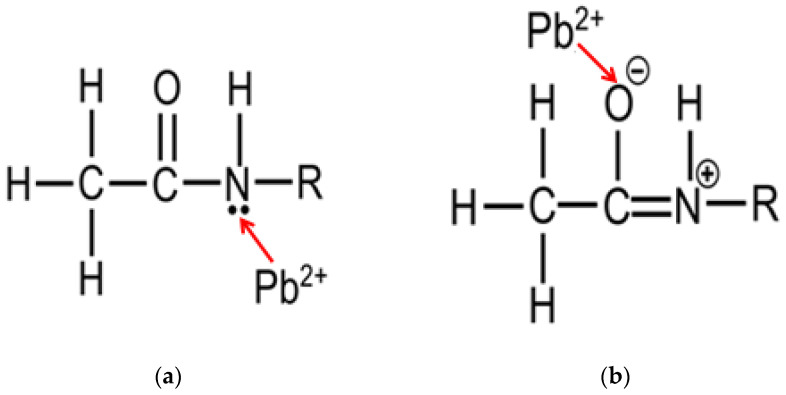
Schematic representation of (**a**) type I and (**b**) type II Pb(II)–amide complexations.

The resonance structure of an amide group has two possible configurations [41], which are named type I and type II. For a type I configuration (refer to Figure 7a), the lone pair of electrons on the N atom is not involved in the conjugation with the carbonyl group, making the N atom relatively electronegative. Hence, a positively charged metal ion (here is Pb^2+^) can coordinate with the N atom of the amide group. Such coordination would result in a positive shift of the carbonyl infrared adsorption to a higher frequency [42]. For a type II configuration (refer to Figure 7b), the lone pair of electrons on the N atom is delocalized into the carbonyl group, making the O atom of the carbonyl group more electronegative. Therefore, a positively charged metal ion can also coordinate with the O atom of the amide group, which would bring about a negative shift of the carbonyl infrared adsorption to a lower frequency [42]. As illustrated in Figure 6b, peak 2′ with a positive shift of ~12 cm^−1^ with respect to peak 2 could be due to the vibration of the Pb(II)-N ligand, which corresponds to the type I complexation. On the other hand, peak 2″ with a negative shift of ~62 cm^−1^ with respect to peak 2 could be due to the vibration of the Pb(II)-O ligand, corresponding to the type II complexation. Based on the experimental observations, it is highly possible that both nitrogen and the carbonyl oxygen of the amide group of the HAMA hydrogel may be simultaneously involved in the formation of the Pb(II)–amide complex.

Despite the contribution from nitrogen, as well as carbonyl oxygen, in the process of the complex formation, the tendency to form a Pb(II)-O ligand seems much higher than that of a Pb(II)-N ligand if one compares the negative shift with the positive one. The negative shift (~62 cm^−1^) is 5 times larger than the positive shift (~12 cm^−1^), suggesting that the carbonyl oxygen of the amide group could play the dominant role in the construction of the Pb(II)–amide complex. This could be explained by the fact that the type II configuration of an amide group is more stable than the type I configuration [41]. Owing to the delocalization, the electron density distributed on the carbonyl oxygen is much greater than that on the nitrogen, making the carbonyl oxygen behave as though it is being completely negatively charged, thereby attracting more Pb(II) ions.

### 3.5. Validation of the Electrochemical Accumulation

**Figure 8:**
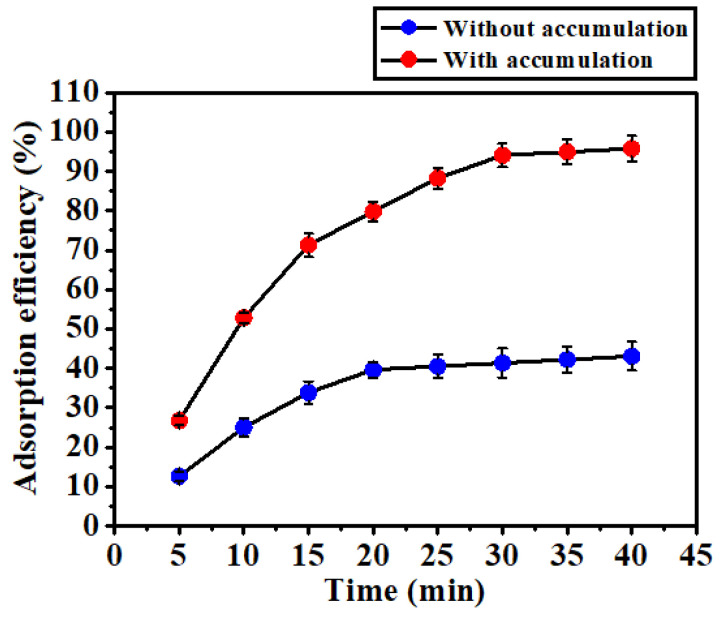
Comparison of adsorption efficiency for the HAMA hydrogel-modified devices without (blue dots) and with (red dots) electrochemical accumulation. Data obtained using three devices.

The effect of electrochemical accumulation pertaining to the proposed adsorption mechanism was investigated by quantifying the amount of Pb(II) ions adsorbed by the modified device. Originally, the Pb(II) concentration of each test solution was 100 µg/L. The concentration was subsequently measured by the ICP-MS instrument after every 5 min of adsorption. The results obtained are presented in Figure 8. When the electrochemical accumulation was not activated, i.e., only the HAMA hydrogel was responsible for the adsorption of Pb(II) ions (blue dots in Figure 8), the adsorption rate slowly increased within the first 20 min. Thereafter, there was no significant increase in the adsorption rate. The adsorption behavior of the HAMA hydrogel could be attributed to the diffusion effect. Initially, metal ions moved into the vicinity of the hydrogel driven by the high-ion-concentration gradient established between the solution and the hydrogel. This corresponded to the increase in the adsorption rate observed at 5, 10, 15, and 20 min. Thereafter, the concentration gradient was significantly reduced, which could have resulted in a rapid saturation of adsorption for the HAMA hydrogel, corresponding to the data points observed at 25, 30, 35, and 40 min. It was calculated that only 43.05% of Pb(II) ions were adsorbed by the device if electrochemical accumulation was not triggered.

On the other hand, when the electrochemical accumulation was activated (red dots in Figure 8), the adsorption rate continuously increased up to 30 min. In addition, the slope of the increase was significantly higher than in the case where electrochemical accumulation was not involved. It was found that 94.08% of Pb(II) ions were adsorbed by the device within 30 min once the electrochemical accumulation had been triggered. These experimental results confirm that electrochemical accumulation is particularly beneficial in facilitating the adsorption rate for the device by maintaining a high concentration gradient between the solution and the hydrogel. Considering the above discussion, it is apparent that both intermolecular complexation and electrochemical accumulation make a significant contribution to the adsorption capability of the HAMA hydrogel-modified electrochemical devices. A comparison of the Pb(II) removal efficiency of the HAMA hydrogel-modified electrochemical device and other polymeric adsorbents is shown in Table 2. Based on the data presented in Table 2, it can be observed that the developed HAMA hydrogel-modified electrochemical device is capable of effectively removing Pb(II) ions with a short adsorption time of 30 min.

### 3.6. Applicability of the HAMA Hydrogel-Modified Device

**Figure 9:**
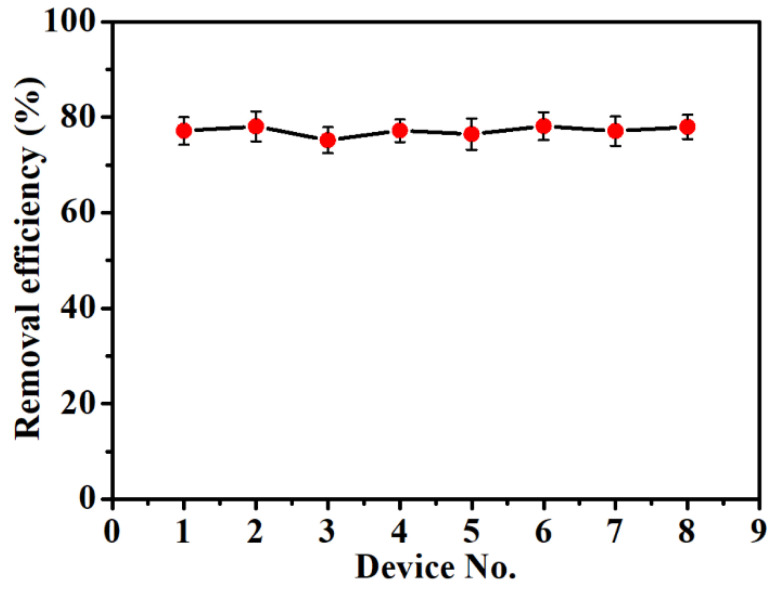
Removal efficiency measured for eight HAMA hydrogel-modified electrochemical devices in a solution of tap water with 400 µg/L Pb(II).

To explore the practical application of the HAMA hydrogel-modified electrochemical device, adsorption experiments were performed in a solution of tap water with 400 µg/L Pb(II). Each device was immersed in the test solution for 20 min, during which the electrochemical accumulation was activated. As shown in Figure 9, all the devices exhibited comparable Pb(II) removal capabilities with an average removal efficiency of 77.2%. The relative standard deviation (RSD) among the eight devices was calculated to be as low as 1.28%, demonstrating good repeatability and reproducibility of the devices. These experimental results reveal that the HAMA hydrogel-functionalized electrochemical device has great potential for deployment at a variety of locations for in situ removal of Pb(II) ions. It is worth mentioning that the water quality conditions (such as pH value, quantity of suspended particulates, other heavy metal ions, etc.) at the locations of interest could affect the adsorption performance of the HAMA hydrogel-modified electrochemical device. Therefore, it is recommended to conduct some preliminary experiments in some specific areas before the large-scale deployment of the devices.

## 4. Conclusions

In this work, an electrochemical device with a three-electrode configuration covered by a layer of HAMA hydrogel was developed for in situ adsorption of Pb(II) ions in aqueous solutions. The devices were successfully fabricated using microfabrication technology along with a surface modification approach. The material properties of the synthesized HAMA hydrogels in the aspects of the swelling capacity, microscopic structure, as well as molecular composition, were systematically evaluated. Based on the analytical outcomes of the fabricated HAMA hydrogel-modified devices, an adsorption mechanism associated with a combined effect of molecular interaction and electrochemical accumulation, which could explain the observed experimental results, was proposed. By performing FTIR analysis on the Pb(II)-soaked HAMA hydrogel, the molecular interaction was corroborated to be the Pb(II)-amide complexation. We found that both nitrogen and the carbonyl oxygen of the amide group were responsible for the formation of the complex, though the carbonyl oxygen could play a dominant role in the course of intermolecular complexation. The contribution of electrochemical accumulation to the adsorption capability of the HAMA hydrogel-modified devices was also confirmed by activating/deactivating the deposition potential applied to the working electrode of the devices. The experimental investigation shows that 94.08% of Pb(II) ions present in the solution can be adsorbed by the device within 30 min. Application of the HAMA hydrogel-modified devices for removing Pb(II) ions in tap water reveals its potential for use for the rapid remediation of Pb(II) contamination. This study paves the way for the design of compact and portable electrochemical devices for in situ removal of Pb(II) ions. Future work will focus on exploring the HAMA hydrogel-modified devices for the simultaneous adsorption of multiple heavy metal ions and employing other validation methods to further investigate the molecular interaction between HAMA hydrogels and different kinds of heavy metal ions.

## Figures and Tables

**Table 1:** Comparison of peak positions in terms of wavenumber (cm^−1^) between the pure HAMA hydrogel and the Pb(II)-soaked HAMA hydrogel.

Peak No.	HAMA (cm^−1^)	Assignment	Peak No.	HAMA with Pb(II) (cm^−1^)	Assignment
1	3436.34	O-H or N-H	1	3430.24	O-H or N-H
2	1628.07	C=O	2′	1640.03	Pb(II)-N
			2″	1565.72	Pb(II)-O
3	1410.64	O-C=O	3	1413.62	O-C=O
4	1153.41	C-O	4	1155.69	C-O
5	1046.36	C-O-C	5	1049.44	C-O-C
6	650.68	bending	6	644.68	bending
			7	1237.61	C-O or C-N

**Table 2:** Comparison of Pb(II) removal efficiency of different adsorbents.

Adsorbent	Removal Efficiency (%)	Adsorption Time (min)	Reference
Poly(allylamine-co-methacrylamide-co-acrylic acid) cryogel	83.54	720	Kim et al. [22]
Poly(*N*,*N*-dimethylacrylamide-co-2-hydroxyethyl methacrylate) copolymer	80.00	300	Ramos-Jacques et al. [23]
Melamine-based crosslinked polyamine/CNT composite	98.63	360	Al Hamouz et al. [25]
Polyisoprene-*b*-polystyrene-*b*-poly(*N*,*N*-dimethyl-acrylamide) polymer	94.80	480	Weidman et al. [27]
Nanochitosan/polyurethane/polypropylene glycol	95.00	60	Saranya et al. [28]
Chitosan-aminopropylsilane graphene oxide nanocomposite hydrogel	82.30	60	Amiri et al. [43]
Copolymerized starch-based hydrogel	87.00	60	Aniagor et al. [44]
Thiol-functionalized silica microsphere-loaded polymeric hydrogel	97.00	1440	Singh et al. [45]
HAMA hydrogel-modified electrochemical device	94.08	30	This work

## Data Availability

Data are available upon reasonable request to Nan Wang (wang_nan@dlut.edu.cn).

## Supplementary Materials

The following supporting information can be downloaded at: https://www.mdpi.com/article/10.3390/bios12090714/s1, Figure S1: Schematic drawing to show detailed dimensions of the polycarbonate mold; Figure S2: The weight of the synthesized HAMA hydrogel samples measured before and after swelling in ultrapure water; Figure S3: Photograph of the synthesized HAMA hydrogel samples upon reaching the equilibrium state of swelling.

## References

[1] Counter S.A., Buchanan L.H., Ortega F. (2012). Association of hemoglobin levels and brainstem auditory evoked responses in lead-exposed children. Clin. Biochem..

[2] Roy A., Hu H., Bellinger D.C., Mukherjee B., Modali R., Nasaruddin K., Schwartz J., Wright R.O., Ettinger A.S., Palaniapan K. (2011). Hemoglobin, lead exposure, and intelligence quotient: Effect modification by the DRD2 Taq IA polymorphism. Environ. Health Perspect..

[3] Philip A., Marsden M.D. (2003). Increased body lead burden―cause or consequence of chronic renal insufficiency?. N. Engl. J. Med..

[4] Bellinger D.C. (2004). Lead. Pediatrics.

[5] Kordas K., Queirolo E.I., Ettinger A.S., Wright R.O., Stoltzfus R.J. (2010). Prevalence and predictors of exposure to multiple metals in preschool children from Montevideo, Uruguay. Sci. Total Environ..

[6] Roy A., Ettinger A.S., Hu H., Bellinger D., Schwartz J., Modali R., Wright R.O., Palaniappan K., Balakrishnan K. (2013). Effect modification by *transferrin* C2 polymorphism on lead exposure, hemoglobin levels, and IQ. Neurotoxicology.

[7] Hwang J.H., Wang X., Zhao D., Rex M.M., Cho H.J., Lee W.H. (2019). A novel nanoporous bismuth electrode sensor for *in situ* heavy metal detection. Electrochim. Acta.

[8] Wang N., Kanhere E., Miao J., Triantafyllou M.S. (2016). Miniaturized chemical sensor with bio-inspired micropillar working electrode array for lead detection. Sens. Actuators B Chem..

[9] Li M., Li Z., Liu C., Chang Y., Wen J., Zhao H., Cao H., Zhang Y., Liu D. (2016). Amino-modification and successive electro-chemical reduction of graphene oxide for highly sensitive electrochemical detection of trace Pb^2+^. Carbon.

[10] Zhou G., Chang J., Cui S., Pu H., Wen Z., Chen J. (2014). Real-time, selective detection of Pb^2+^ in water using a reduced graphene oxide/gold nanoparticle field-effect transistor device. ACS Appl. Mater. Interfaces.

[11] Niu X., Zhong Y., Chen R., Wang F., Liu Y., Luo D. (2018). A “turn-on” fluorescence sensor for Pb^2+^ detection based on graphene quantum dots and gold nanoparticles. Sens. Actuators B Chem..

[12] Saha S.K., Ghosh K.R., Gao J.P., Wang Z.Y. (2014). Highly sensitive dual-mode fluorescence detection of lead ion in water using aggregation-induced emissive polymers. Macromol. Rapid Commun..

[13] Zhan S., Wu Y., Liu L., Xing H., He L., Zhan X., Luo Y., Zhou P. (2013). A simple fluorescent assay for lead(II) detection based on lead(II)-stabilized G-quadruplex formation. RSC Adv..

[14] Skotadis E., Tsekenis G., Chatzipetrou M., Patsiouras L., Madianos L., Bousoulas P., Zergioti I., Tsoukalas D. (2017). Heavy metal ion detection using DNAzyme-modified platinum nanoparticle networks. Sens. Actuators B Chem..

[15] Huang Y., Ma Y., Chen Y., Wu X., Fang L., Zhu Z., Yang C.J. (2014). Target-responsive DNAzyme cross-linked hydrogel for visual quantitative detection of lead. Anal. Chem..

[16] Wang Y., Irudayaraj J. (2011). A SERS DNAzyme biosensor for lead ion detection. Chem. Commun..

[17] Renfrew D. (2018). Lead poisoning and the dangers of pragmatism. Int. J. Environ. Res. Public Health.

[18] Chowdhury S., Mazumder M.A.J., Al-Attas O., Husain T. (2016). Heavy metals in drinking water: Occurrences, implications, and future needs in developing countries. Sci. Total Environ..

[19] Gupta V.K., Agarwal S., Saleh T.A. (2011). Synthesis and characterization of alumina-coated carbon nanotubes and their ap-plication for lead removal. J. Hazard. Mater..

[20] Foo K.Y., Hameed B.H. (2010). Insights into the modeling of adsorption isotherm systems. Chem. Eng. J..

[21] Liu Y., Zhang W., Zhao C., Wang H., Chen J., Yang L., Feng J., Yan W. (2019). Study on the synthesis of poly(pyrrole methane)s with the hydroxyl in different substituent position and their selective adsorption for Pb^2+^. Chem. Eng. J..

[22] Kim M.Y., Lee T.G. (2019). Removal of Pb(II) ions from aqueous solutions using functionalized cryogels. Chemosphere.

[23] Ramos-Jacques A.L., Lujan-Montelongo J.A., Silva-Cuevas C., Cortez-Valadez M., Estevez M., Hernandez-Martinez A.R. (2018). Lead(II) removal by poly(*N*,*N*-dimethylacrylamide-*co*-2-hydroxyethyl methacrylate). Eur. Polym. J..

[24] Mohammadinezhad A., Marandi G.B., Farsadrooh M., Ja-vadian H. (2018). Synthesis of poly(acrylamide-co-itaconic ac-id)/MWCNTs superabsorbent hydrogel nanocomposite by ultra-sound-assisted technique: Swelling behavior and Pb(II) adsorption capacity. Ultrason. Sonochem..

[25] Al Hamouz O.C.S., Adelabu I.O., Saleh T.A. (2017). Novel cross-linked melamine based polyamine/CNT composites for lead ions removal. J. Environ. Manag..

[26] Zhang Y., Li Z. (2017). Heavy metals removal using hydrogel-supported nanosized hydrous ferric oxide: Synthesis, characterization, and mechanism. Sci. Total Environ..

[27] Weidman J.L., Mulvenna R.A., Boudouris B.W., Phillip W.A. (2017). Nanoporous block polymer thin films functionalized with bio-inspired ligands for the efficient capture of heavy metal ions from water. ACS Appl. Mater. Interfaces.

[28] Saranya M., Latha S., Reddi M.R.G., Gomathi T., Sudha P.N., Anil S. (2017). Adsorption studies of Lead(II) from aqueous solution onto Nanochitosan /Polyurethane /Polypropylene glycol ternary blends. Int. J. Biol. Macromol..

[29] Chandrasekharan A., Seong K.Y., Yim S.G., Kim S., Seo S., Yoon J., Yang S.Y. (2019). In situ photocrosslinkable hyaluronic ac-id-based surgical glue with tunable mechanical properties and high adhesive strength. J. Polym. Sci. A Polym. Chem..

[30] Ren L., Lim Y.T. (2018). Degradation-regulatable architectured implantable macroporous scaffold for the spatiotemporal modulation of immunosuppressive microenvironment and enhanced combination cancer immunotherapy. Adv. Funct. Mater..

[31] Yang G., Fu S., Yao W., Wang X., Zha Q., Tang R. (2017). Hyaluronic acid nanogels prepared via ortho ester linkages show pH-triggered behavior, enhanced penetration and antitumor efficacy in 3-D tumor spheroids. J. Colloid Interface Sci..

[32] Deng X., Korogiannaki M., Rastegari B., Zhang J., Chen M., Fu Q., Sheardown H., Filipe C.D.M., Hoare T. (2016). “Click” chemistry-tethered hyaluronic acid-based contact lens coatings improve lens wettability and lower protein adsorption. ACS Appl. Mater. Interfaces.

[33] Tao K., Yi H., Yang Y., Chang H., Wu J., Tang L., Yang Z., Wang N., Hu L., Fu Y. (2020). Origami-inspired electret-based triboelectric generator for biomechanical and ocean wave energy harvesting. Nano Energy.

[34] Smeds K.A., Pfister-Serres A., Miki D., Dastgheib K., In-oue M., Hatchell D.L., Grinstaff M.W. (2001). Photocrosslinkable poly-saccharides for *in situ* hydrogel formation. J. Biomed. Mater. Res..

[35] Magalhaes J., Sousa R.A., Mano J.F., Reis R.L., Blanco F.J., Roman J.S. (2013). Synthesis and characterization of sensitive hydrogels based on semi-interpenetrated networks of poly[2-ethyl-(2-pyrrolidone) methacrylate] and hyaluronic acid. J. Biomed. Mater. Res. Part A.

[36] Anirudhan T.S., Nair S.S., Nair A.S. (2016). Fabrication of a bioadhesive transdermal device from chitosan and hyaluronic acid for the controlled release of lidocaine. Carbohydr. Polym..

[37] Reddy K.J., Karunakaran K.T. (2013). Purification and characterization of hyaluronic acid produced by *Streptococcus zooepidemicus* strain 3523-7. J. BioScience Biotechnol..

[38] Wang N., Kanhere E., Miao J., Triantafyllou M.S. (2018). Nanoparticles-modified chemical sensor fabricated on a flexible polymer substrate for cadmium(II) detection. Polymers.

[39] Lee S., Park S.K., Choi E., Piao Y. (2016). Voltammetric determination of trace heavy metals using an electrochemically deposited graphene/bismuth nanocomposite film-modified glassy carbon electrode. J. Electroanal. Chem..

[40] Serrano N., Alberich A., Diaz-Cruz J.M., Arino C., Esteban M. (2013). Coating methods, modifiers and applications of bismuth screen-printed electrodes. Trends Anal. Chem..

[41] Clayden J., Greeves N., Warren S. (2012). Organic Chemistry.

[42] Feng Y., Schmidt A., Weiss R.A. (1996). Compatibilization of polymer blends by complexation. 1. spectroscopic characterization of ion-amide interactions in ionomer/polyamide blends. Macromolecules.

[43] Amiri S., Asghari A., Vatanpour V., Rajabi M. (2021). Fabrication of chitosan-aminopropylsilane graphene oxide nanocomposite hydrogel embedded PES membrane for improved filtration performance and lead separation. J. Environ. Manag..

[44] Aniagor C.O., Afifi M.A., Hashem A. (2022). Rapid and efficient uptake of aqueous lead pollutant using starch-based superabsorbent hydrogel. Polym. Bull..

[45] Singh S., Basu H., Bassan M.K.T., Singhal R.K. (2022). Thiol functionalised silica microsphere loaded polymeric hydrogel: Development of a novel hybrid sorbent for removal of lead and cadmium. Chemosphere.

