# Supplementary material for: Hyaluronic Acid Methacrylate Hydrogel-Modified Electrochemical Device for Adsorptive Removal of Lead(II)
Source: Biosensors (Basel). 2022 Sep 2;12(9):714. doi: 10.3390/bios12090714 (PMC9496323; doi:10.3390/bios12090714)
Supplement: Supplementary file 1 [file biosensors-12-00714-s001.zip › biosensors-1857404-supplementary.pdf]

Hyaluronic Acid Methacrylate Hydrogel-Modified Electrochemical Device for Adsorptive Removal of Lead(II)

Nan Wang[1,2,*], Meghali Bora[2], Song Hao[3,*], Kai Tao[4], Jin Wu[5], Liangxing Hu[6], Jianjun Liao[7], Shiwei Lin[8], Michael S. Triantafyllou[2,9], Xiaogan Li[1]

[1]School of Microelectronics, Dalian University of Technology, Dalian, 116024, PR China
[2]Center for Environmental Sensing and Modeling (CENSAM) IRG, Singapore-MIT Alliance for Research and Technology (SMART) Centre, 138602, Singapore
[3]School of Optoelectronic Engineering and Instrumentation Science, Dalian University of Technology, Dalian, 116024, PR China
[4]Ministry of Education Key Laboratory of Micro and Nano Systems for Aerospace, School of Mechanical Engineering, Northwestern Polytechnical University, Xi'an, 710072, PR China
[5]State Key Laboratory of Optoelectronic Materials and Technologies and the Guangdong Province Key Laboratory of Display Material and Technology, School of Electronics and Information Technology, Sun Yat-sen University, Guangzhou, 510275, PR China
[6]School of Electrical and Electronic Engineering, Nanyang Technological University, 639798, Singapore
[7]Key Laboratory of Agro-Forestry Environmental Processes and Ecological Regulation of Hainan Province, School of Ecological and Environmental Sciences, Hainan University, Haikou, 570228, PR China
[8]School of Materials Science and Engineering, Hainan University, Haikou, 570228, PR China
[9]Department of Mechanical Engineering, Massachusetts Institute of Technology, Cambridge, MA 02139, USA
[*]Correspondence: N. Wang, wang_nan@dlut.edu.cn; S. Hao, hao@dlut.edu.cn

This file contains all the supplementary figures. The detailed description of each figure is illustrated in the main content of the manuscript.

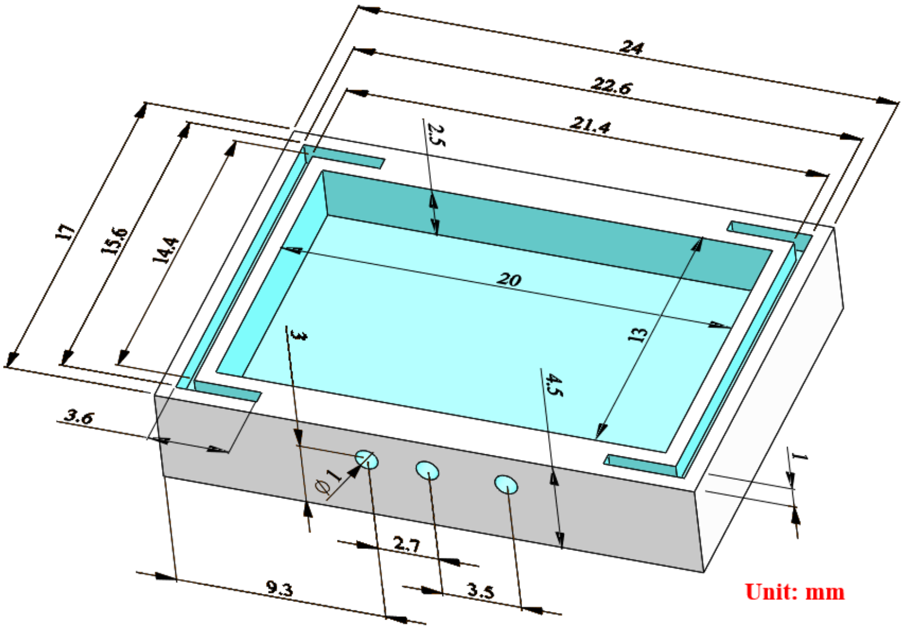

Figure S1. Schematic drawing to show detailed dimensions of the polycarbonate mold.

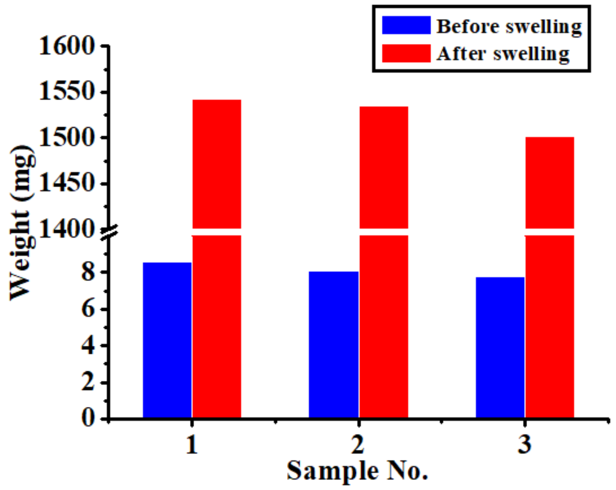

Figure S2. The weight of the synthesized HAMA hydrogel samples measured before and after swelling in ultrapure water.

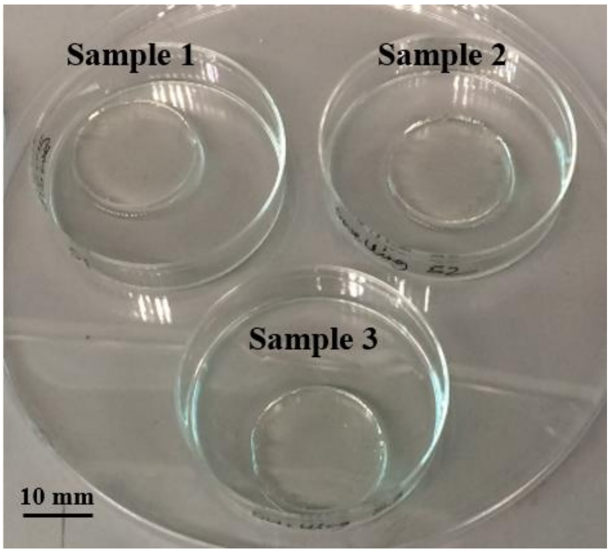

Figure S3. Photograph of the synthesized HAMA hydrogel samples upon reaching the equilibrium state of swelling.
